# Cancer comes of age embryonically

**DOI:** 10.18632/aging.100959

**Published:** 2016-05-05

**Authors:** Vi K. Chiu, Anne-France Le Rolle

**Affiliations:** Division of Hematology/Oncology, Department of Medicine, University of California, Irvine, CA 92697, USA

**Keywords:** embryonic stem cell, KRAS, colon cancer initiation

The incidence of cancer increases exponentially with age. Aging may lead to pathologies due to the physiologic decline or aberrancy in the functions of adult stem cells, whose intrinsic capacity for self-renewal helps maintain tissue homeostasis. The majority of cancers arise from replication-induced somatic mutations in adult stem cells [1], which predisposes to hijacking their self-renewal pathways to drive tumor development. Yet the modulation of stem cell signaling by oncogenic driver mutations in cancer initiation is not well characterized. The hierarchically structured colon epithelium presents an ideal *in vivo* cancer model to assess the step-wise malignant transformation of crypt base colon stem cells into colon adenoma and carcinoma cells.

Hitherto, the initiation of colon cancer has been attributed mainly to the aberrant activation of WNT signaling as a result of *APC* or *CTNNB1* mutations, which are found in >90% of early colon adenomas [2]. Indeed, it has been suggested that the development of oncogenic KRAS mutation (KRAS^mut^) in late colon adenomas drive their malignant transformation to cancer by further promoting WNT activation through β-catenin nuclear localization [3]. However, aberrant WNT signaling *per se*, even when targeted to murine colon stem cells to constitutively activate their intestinal stem cell program, was capable of initiating colon adenomas but not invasive colon cancers [4]. Nearly all colon cancers originate from colon adenomas, yet ≤ 5% of colon adenomas ultimately transform into colon cancers [5]. Thus the biologic programs driving colon cancer initiation, which is best defined as the direct malignant transformation of colon adenomas into stage I colon cancers, are distinct from the premalignant activated WNT program that is responsible for colon adenoma initiation.

We have found that embryonic stem cell-like program activation is a major mechanistic driver of colon cancer initiation, and the presence of KRAS^mut^ further increases this tumorigenic and dedifferentiation program [6]. The ability of KRAS^mut^ to dedifferentiate *APC* mutated colon adenoma cells depends on their moderate plasticity, since KRAS^mut^ by itself in the cellular context of differentiated epithelial cells cannot induce the embryonic stem cell-like program [6, 7]. We have proposed an *in vivo* plasticity model in which the achievement of high plasticity, as exemplify by KRAS^mut^ mediated induction of the embryonic stem cell-like program in colon adenomas, optimizes colon cancer initiation. Currently, there are two conflicting theories for the colon cancer cells of origin. The top-down theory requires that differentiated crypt top colon cells undergo dedifferentiation to become the colon cancer cells of origin. The bottom-up theory suggests that crypt base WNT activated colon stem cells, which give rise to colon adenomas, are the colon cancer cells of origin [4]. However, human colon cancers occur years to decades after the development of colon adenomas. It is more precise to identify the colon cancer cells of origin as the colon adenoma cells, which are also long-lived and accumulate tumorigenic driver mutations with aging. Unlike colon stem cells, colon adenoma cells are not restricted to the crypt base, and their propagation by crypt fission and random expansion into neighboring normal crypts accounts for their crypt top presence. Our *in vivo* plasticity model helps explain the paradoxical findings that lend support to both the top-down and bottom-up theories on the colon cancer cells of origin.

Intriguingly, embryonic stem cells arise at the beginning of human ontogeny and cancer cells arise dramatically toward the end. Thus cancer cells have come nearly full circle in reactivating the embryonic stem cell-like program, which imparts greater phenotypic plasticity to thrive with environmental variations. Cancers may be the unfortunate failed manifestations of nature's quest for the fountain of youth. Nevertheless, the inhibition of the embryonic stem cell-like program offers novel therapeutic strategies to prevent cancer and inhibit KRAS^mut^ tumors.
